# Global tissue-specific transcriptome analysis of *Citrus sinensis* fruit across six developmental stages

**DOI:** 10.1038/s41597-019-0162-y

**Published:** 2019-08-21

**Authors:** Guizhi Feng, Juxun Wu, Hualin Yi

**Affiliations:** Key Laboratory of Horticultural Plant Biology, Ministry of Education, Huazhong Agricultural University, Wuhan, 430070 China

**Keywords:** Plant molecular biology, Plant development

## Abstract

*Citrus sinensis* fruit is a type of nonclimacteric fruit that mainly consists of four tissues: the epicarp, albedo, segment membrane and juice sac. The fruit quality is determined by the characteristics of these four tissues. However, our knowledge of the molecular processes that occur in these four tissues during citrus fruit development and ripening is limited. Tissue-specific transcriptomes provide a comprehensive and detailed molecular regulatory network of citrus fruit development and ripening. In our study, we collected four types of tissue from *C*. *sinensis* fruits at six developmental stages. A total of 72 libraries were constructed from 24 samples (each sample had three replicates), and the transcriptomes were sequenced by an Illumina HiSeq 4000. The comprehensive analyses of the transcriptomes from the four tissues and six developmental stages presented here provide a valuable resource for the discovery of the molecular networks underlying citrus fruit development and ripening.

## Abstract (summary)

**Table:** 

Design Type(s)	transcription profiling design • organism part comparison design • organism development design
Measurement Type(s)	transcription profiling assay
Technology Type(s)	RNA sequencing
Factor Type(s)	biological replicate • fruit part
Sample Characteristic(s)	Citrus sinensis • exocarp • albedo • membrane organ • juice sac tissue

Machine-accessible metadata file describing the reported data (ISA-Tab format)

## Background & Summary

Citrus, a nonclimacteric fruit, is widely cultivated worldwide. Citrus is mainly composed of the inedible epicarp (EP) and albedo (AL) and the edible segment membrane (SM) and juice sac (JS). The development and ripening of citrus fruit is a complex and sophisticated regulatory process that can be divided into three stages: cell division stage; expansion stage involving cell enlargement and water, sugar accumulation; and ripening stage^1^. At present, most studies investigating citrus fruit development and ripening are based on the organ-wide or mixed-tissue level, which inevitably obscures many tissue-specific phenomena. Therefore, a systematic study of the different tissues and periods within the same citrus fruit can help us to more clearly understand the developmental characteristics of different tissues and their interrelationships.

The analysis of tissue-specific expression profiles avoids the potential dilution of location-specific regulation and has been proven to effectively reveal otherwise undetectable biological pathways and regulatory networks^2^. In recent years, tissue-specific transcriptomes have been used in many types of plants. Through the transcriptome analysis of multiple tissues in young tomato plants, the unique developmental regulation of tomato seed was discovered^3^. The association between the transcriptome of 23 tissues of cucumber and volatile organic compounds in cucumber was screened to select a group of candidate genes that may be involved in the synthesis of volatile substances in cucumber^4^. Through the transcriptome analysis of different structures of maize seed, the key role of MRP-1 (MYB-Related Protein-1) in maize endosperm development was found^5^. By analyzing the transcriptome data of five developmental stages and five tissues of strawberry, it was found that the role of the endosperm and seed coat in auxin and gibberellin biosynthesis for fruit set is more prominent than that of the embryo^6^. The mechanisms underlying fruit development and ripening are unique among different fruit types. A comprehensive transcriptome profile of the tissue types and stage types can reveal the enormous diversity in gene expression associated with tissue type and developmental stage. Anatomically, we usually divide citrus into four tissues: EP, AL, SM, and JS. Different tissues play different roles in the development and maturation of citrus fruits. The development of the EP determines the appearance of the fruit, and the metabolism of carotenoids plays an important role in this process^7,8^; the development of the AL determines how easy it is to peel the fruit, the hardness of the fruit, etc. SM directly determines the chewiness of the fruit, and cell wall metabolism plays an important role in this process^9^; the JS stores large amounts of sugars and organic acids. Sucrose enters the JS after unloading at the SM phloem^10,11^. A large amount of organic acid is accumulated in the JS, 90% of which is citric acid^12^. Currently, our understanding of the underlying molecular mechanisms involved in these processes is limited.

**Fig. 1:**
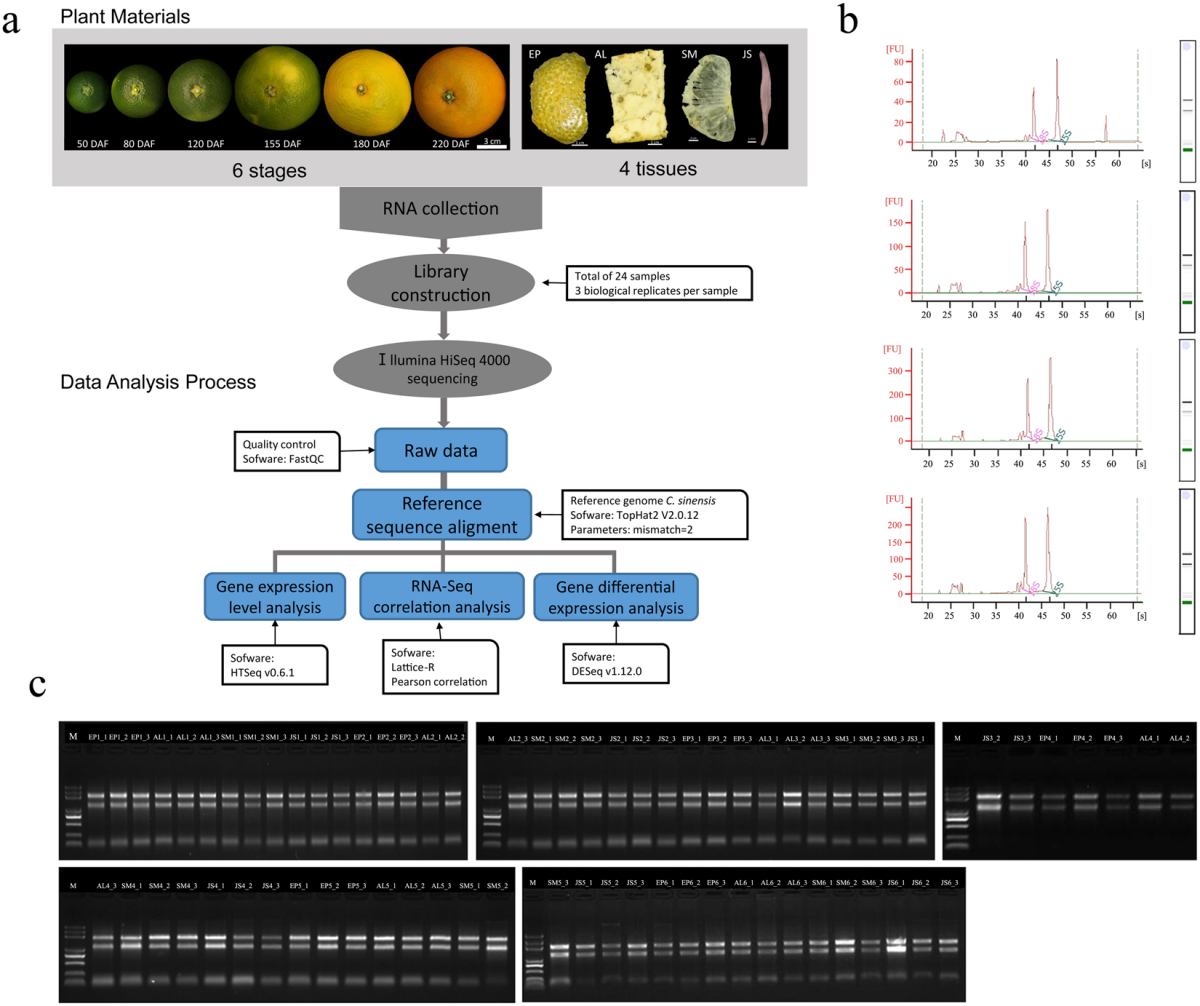
Study work flow from sample preparation through sequence processing. (**a**) Schematic overview of the study. (**b**) Presequencing RNA quality control check with a Bioanalyzer 2100 system, and example results for EP, AL, SM, and JS samples show good quality RNA for library preparation. (**c**) RNA from 72 tissues analyzed by agarose gel electrophoresis. M, Marker DL2000. In all figures that follow, the samples are named “Tissue_stage_replicate” or “Tissue_stage”. EP_1_1 means replicate 1 of the epicarp at stage 1. AL_2 means the albedo sample at stage 2.

In this study, we performed transcriptome sequencing of four tissues (EP, AL, SM and JS) throughout the development and ripening stages (50, 80, 120, 155, 180 and 220 days after flowering (DAF)) of the navel orange fruit. After we removed low-quality sequencing data, we obtained a total of 549 Gb (G bases) of transcriptome data from the collected samples (no less than 6 Gb per sample). We describe how we collected and processed samples, extracted mRNA, built a cDNA library, controlled the quality of transcriptome data, aligned the data to a reference genome, and performed correlation analysis between samples. Finally, we describe how we obtained reliable data for use in subsequent analysis and future research. All the experimental processes involved in the paper are shown in Fig. 1a.

## Methods

### Overview of experimental design

Fruit samples of the ‘Fengjie 72-1’ navel orange (*C*. *sinensis* L. Osbeck) were harvested after the second physiological fruit-falling period (50 DAF), expansion period (80, 120 and 155 DAF), coloring period (180 DAF) and full-ripening period (220 DAF). Four fruit tissues (EP, AL, SM and JS) were manually dissected at every stage, rapidly frozen in liquid nitrogen and kept at −80 °C. The experimental design and analysis pipeline are shown in Fig. 1a. After the extraction of the total RNA, a total of 72 libraries were constructed from 24 samples (each sample had three replicates). A total of 72 transcriptome profiles were obtained by RNA-seq using an Illumina HiSeq^TM^ 4000 sequencing platform. Subsequently, the clean reads filtered from the raw reads were mapped to the reference genome of *C*. *sinensis*^13,14^ (http://citrus.hzau.edu.cn/orange/index.php). The gene expression analyses were performed with HTSeq^15^, and the differential expression analyses were performed with DESeq2^16,17^.

### Sample collection

We selected a total of 9 trees (3 trees as one biological replicate) to collect samples. These nine trees were grafted on the same rootstock *Poncirus trifoliata* (L.) Raf and cultivated in the same orchard (Fengjie, Chongqing City, China). At each stage, four fruits were sampled from each tree, and a total of 12 fruits were mixed as one biological replicate for each sample. After the fruit was sampled from the tree, the EP was quickly cleaned with distilled water and dried with a sterile gauze. Subsequently, the EP was gently scraped with a scalpel. Then, the residual EP was removed, and the AL was separated. This step ensured that there were no residual colored substances or internal tissues on the AL. Finally, the SM and JS were separated. The JS was gently scraped from the SM, and the SM was rinsed with distilled water to remove the residual JS liquid. All of the above anatomical and tissue acquisition steps were rapidly performed in a low temperature environment created by crushed ice, and the separated tissues were quickly treated with liquid nitrogen and stored at −80 °C.

### RNA extraction, library construction, and RNA sequencing

In total, 72 materials (3 biological replicates per sample) were used to extract total RNA, as previously described^18^. In brief, the RNA extraction protocol includes two parts: rough extraction and purification extraction. Rough extraction: approximately 0.5 g sample powder ground in liquid nitrogen is transferred to a 10 ml centrifuge tube with 5 ml Trizol Buffer; add 3 ml chloroform, shake 30 s, then stand at room temperature for 10 min; subsequently, add an equal volume of pre-cooled isopropanol, mix gently and stand for 10 min; discard the supernatant and soak in 3 ml of pre-cooled 75% ethanol for 1 h; next, centrifuge at 12000 rpm for 4 min at 4 °C, then discard the ethanol and dry it in air. Purification extraction: add 800 ul TESAR into the 10 ml centrifuge tube with RNA precipitate and vortex it; then, add 800 ul AQ/CTAB and 800 ul Bu/CTAB and vortex for 15 min; next, transfer it into two 1.5 ml centrifuge tubes and centrifuge at room temperature for 6 min; the supernatant is transferred to a 1.5 ml centrifuge tube, and add 350 ul NaCl (0.2 mol/L), vortex for 1 min, centrifuge at room temperature for 15 min; after carefully pipette the supernatant into a 1.5 ml centrifuge tube, add 50 ul NaAC (3 mol/L) and 1 ml pre-chilled ethanol, mix gently, and freeze at −20 °C for 1 h; at the end, centrifuge at 13200 rpm for 30 min at 4 °C, discard the supernatant, and dissolve it in 40 ul DEPC water. The quality of RNA was detected by agarose gels (Fig. 1c). RNA purity (OD260/280 ratio) was checked using a NanoPhotometer® spectrophotometer (IMPLEN, CA, USA); RNA concentration was measured using a Qubit® RNA Assay Kit in Qubit® 2.0 Flurometer (Life Technologies, CA, USA). RNA integrity was assessed using a RNA Nano 6000 Assay Kit on a Bioanalyzer 2100 system (Agilent Technologies, CA, USA). Total RNA with RIN ≥6.5 was used for cDNA library construction (Fig. 1b and Online-only Table 1).

A total of 3 μg of RNA per sample was used as the input material to construct the cDNA library. The sequencing libraries generated using NEBNext® Ultra™ RNA Library Prep Kit for Illumina® (NEB, USA) were added to the attribute sequence for each sample according to the manufacturer’s recommendations^19,20^. Briefly, mRNA was purified from total RNA using magnetic beads attached to a poly-T oligo and cleaved using a divalent cation in the NEBNext strand synthesis reaction buffer (5X) in the a first elevated temperature. First-strand cDNA was synthesized using random hexamer primers and M-MuLV reverse transcriptase (RNase H-). Next, second strand cDNA synthesis was performed with DNA polymerase I and RNase H. Exonuclease/endonuclease/polymerase activity joined the protruding end into blunt end. NEBNext 3′ end polyadenylation of the DNA fragments resulted in the hairpin loop structure of the connection adapter in preparation for hybridization. To select cDNA fragments of preferably 150–200 bp in length, the library fragments were purified using the AMPure XP system (Beckman Coulter, Beverly, USA)^21^. The cDNA ligated with the size-selected linker was then ligated with 3 μl of USER enzyme (NEB, USA) for 15 minutes at 37 °C and then at 95 °C for 5 minutes before PCR. Then, polymerase Phusion high-fidelity DNA, universal PCR primers and index (X) primer were added for PCR. Finally, PCR enrichment was performed to obtain a final cDNA library. After the library was constructed, preliminary quantification was performed using Qubit 2.0, and the library was diluted to 1 ng/µl. Then, the insert size of the library was detected using Agilent 2100. After the insert size was verified, Q-PCR was performed. The effective concentration of the library was accurately quantified (effective library concentration >2 nM) to ensure library quality.

The libraries were combined into paired pools, and double-end sequencing was performed by sequencing the cDNA product that was reverse transcribed into a small fragment of 150–200 bp using a HiSeq 4000 instrument (Illumina, San Diego, USA) at Novogene Company (Beijing, China). The raw data files obtained from high-throughput sequencing were converted to the original sequence by CASAVA Base Calling analysis^22^. The results were stored in a FASTQ file.

### Preprocessing of sequencing data

**Fig. 2:**
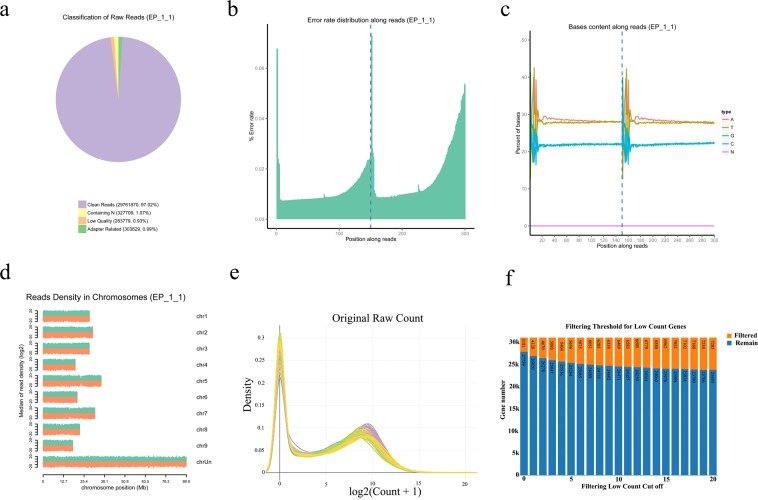
Global assessment of transcriptome data. Example results for (**a**) sequencing error rate distribution, (**b**) A/T/G/C content distribution, and (**c**) composition of raw data, and (**d**) the density distribution of reads on the chromosome, which show the good quality of the transcriptome libraries. (**e**) Distribution density of original raw counts. The x axis is log_2_ (count +1), and the y axis is the density. (**f**) Filtered genes with a total read count below the thresholds. Max threshold = 20.

The raw data obtained by sequencing was filtered by removing the reads with adapters, reads with N (N means that the base information cannot be determined) greater than 10%, and low-quality reads (Qphred <=20 bases account for more than 50% of the entire read length The reads) to get clean reads^23,24^ (Fig. 2a). Then, the quality-controlled reads (clean reads) of the 72 transcriptome libraries were mapped to the reference genome of *C*. *sinensis*^13^. TopHat (v2.0.12)^14^ was used as the mapping tool. First, the entire obtained sequence was aligned to the genomic exon, and the obtained sequence was then segmentally aligned with the two exons of the genome^24^. The statistical comparisons of the reads with the reference genome are shown in Online-only Table 1. Because the reference genome is generated from the dihaploid DNA of sweet orange and the assembled sequence only covers 87.3% of the estimated orange genome^13^, the rates of uniquely mapped reads of 72 navel orange transcriptome libraries ranged from 67.74% to 76.46% are in the normal range. Samtools was used to convert the .*sam* file to a .*bam* file with the default parameters. The count matrix file was imported into DESeq2^16,17^ for differential expression analysis. The Benjamini and Hochberg method was used to adjust the *P* value obtained to control the false discovery rate. The gene expression levels were calculated by the FPKM (Fragments Per Kilobase of transcript per Million mapped reads) method using HTSeq^15^ (v0.6.1).

## Data Records

The RNA-Seq raw data of 72 samples were deposited in NCBI Sequence Read Archive with accession number SRP182638^25^. The file of gene expression level was deposited in NCBI Gene Expression Omnibus (GEO) with accession number GSE125726^26^.

## Technical Validation

### Quality control

The original sequence obtained after sequencing was used for subsequent analysis after a series of quality control analyses. First, the distribution of the sequencing error rate was evaluated (Fig. 2b). The error rate of each sequenced base was determined by the Phred score (Q_phred_) with the formula (1: Q_phred_ = −10 log_10_(e)), and the Phred value is passed through a base calling analysis. The probability model showed that this model can accurately predict the error rate of base discrimination. Subsequently, the distribution of A/T/G/C content was inspected to detect the presence or absence of AT and GC separation (Fig. 2c). At the same time, the Q20, Q30 and GC contents of the clean data were calculated (Online-only Table 1). The clean data obtained by filtering the raw data and the clean reads were compared to the chromosome (Fig. 2d).

**Fig. 3:**
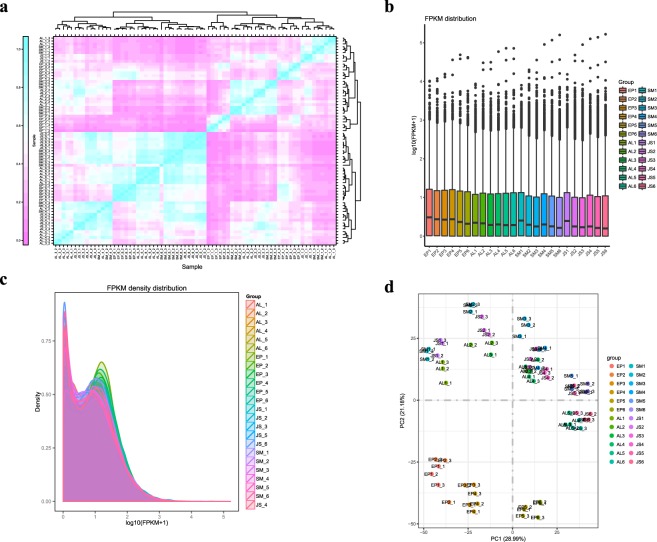
Analysis of global gene expression among fruit tissues. (**a**) Biological replicate sample correlation analysis. (**b**) Boxplot shows different sample FPKM distributions. The y axis is log_10_ (FPKM +1). (**c**) Intuitive display genes expressed at different FPKM levels. The x axis is log_10_ (FPKM +1), and the y axis is the density of the gene. (**d**) Two-dimensional PCA analysis with nonmetric multidimensional scaling (NMDS).

The raw count was homogenized for all samples by log_2_ (count +1), and distribution density statistics were performed (Fig. 2e). The raw counts of all samples were screened with a maximum screening threshold of 20, and the number of genes under different screening thresholds was counted^27,28^ (Fig. 2f). Additionally, the count matrix was clustered to generate clustered heat map with the lattice-R package, and the correlation coefficient was calculated with Pearson correlation. The results showed a good correlation between the three biological replicates of the different samples (Fig. 3a).

### Analysis of RNA-seq data

First, we calculated the FPKM distribution density of all samples (Fig. 3b,c). Additionally, the FPKM value of the gene in the sample was used as the input data for nonmetric multidimensional scaling (NMDS)^29^ to reduce and realign the data in a visual low-dimensional space, which was maximized in a plane scatter plot (Fig. 3d). The x-axis reflects the extent of the relationship between the biological replicates. Moreover, we found that the EP was more independent than the other three tissues, and the differences between tissues were greater than those between developmental period, while AL, SM, and JS showed large differences between periods. The expression levels of individual genes were submitted to NCBI GEO^26^.

**Fig. 4:**
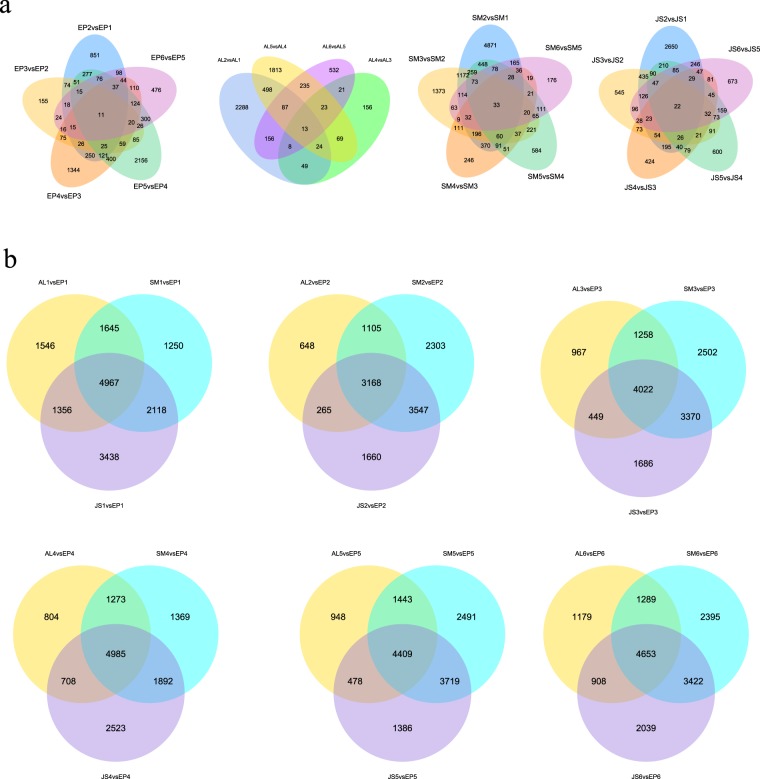
Veen plot shows differentially expressed genes in different tissues at the same stage (**a**) and among different stages in the same tissue (**b**).

**Fig. 5:**
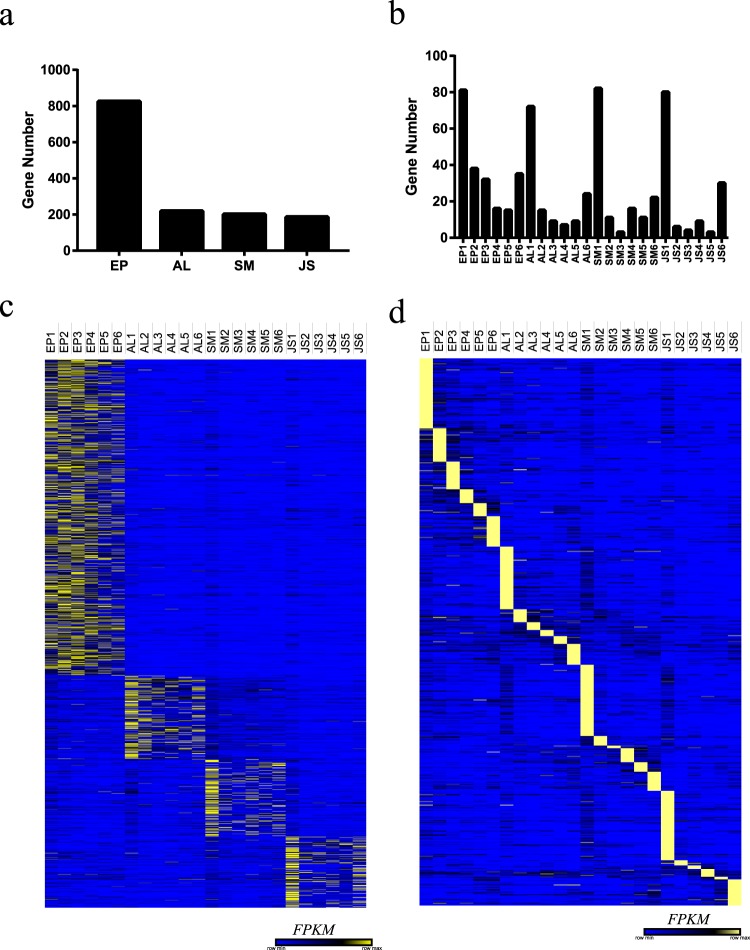
Screening of the spatiotemporal expression genes. (**a**) The number of tissue-specific genes. (**b**) The number of sample-specific genes. (**c**) Heat-map analysis of tissue-specific genes. (**d**) Heat-map analysis of sample-specific genes.

Genes with adjusted *P* values < 0.05 as determined by DESeq2^16,17^ were designated as differentially expressed genes^17,30,31^. The differentially expressed genes in different tissues and different stages during the development of navel orange were screened. The results of spatiotemporal differences in the genes are presented in the form of Venn diagram^32^ (Fig. 4a,b). At different levels, approximately 100 to 5000 differentially expressed genes were screened in the different tissues (Fig. 4a). In the EP, the number of differentially expressed genes identified in the EP5 vs. EP4 comparison was the highest. In contrast, AL, SM and JS appeared to have the largest number of differentially expressed genes between Stage 2 and Stage 1. In particular, we did not screen for differentially expressed genes in AL3 vs. AL2 (Fig. 4a). Furthermore, at the tissue level, we compared the AL, SM, and JS at different developmental stages with EP (EP as a control), and different comparison combinations identified approximately 1000 to 3000 differential genes (Fig. 4b). From the distribution of the number of differential genes, the overall comparison with the EP was JS > SM > AL, which indicates that the spatial distance of the tissue affects the difference in the gene expression of the fruit. A larger spatial distance is correlated with a more obvious the difference between tissues. Finally, we also screened for tissue-specific genes and sample-specific genes. We identified 825 differentially expressed genes in the EP, which is much more than the 219, 201, and 186 genes identified for AL, SM, and JS, respectively (Fig. 5a,b), which further illustrates the specific function of the EP. In addition, the maximum number of all four tissue sample-specific genes was observed in Stage 1 (Fig. 5c,d).

### ISA-Tab metadata file

Download metadata file

## Online-only Table

**Online-only Table 1:** Statistics analyses of the 72 transcriptomes prepared from 24 samples.

Samples	RIN	Library layout	Clean bases	GC(%)	Q30(%)	Total reads	Total mapped	Uniquely mapped	Biosample accession	Tissue
EP_1_1	7.7	Paired	8.93G	44.06	92.43	59523740	45424115 (76.31%)	43462401 (73.02%)	GSM3581015	Epicarp
EP_1_2	7.5	Paired	8.07G	44.01	92.5	53786352	41070563 (76.36%)	39298671 (73.06%)	GSM3581016	Epicarp
EP_1_3	7.1	Paired	8.23G	44.02	92.71	54858752	42326242 (77.15%)	40620785 (74.05%)	GSM3581017	Epicarp
EP_2_1	7.4	Paired	7.37G	44.14	92.27	49110318	37656718 (76.68%)	36197028 (73.71%)	GSM3581018	Epicarp
EP_2_2	8.1	Paired	7.99G	44.48	92.6	53234154	40001604 (75.14%)	38262194 (71.88%)	GSM3581019	Epicarp
EP_2_3	7	Paired	7.47G	44.62	92.57	49815492	37654138 (75.59%)	36130331 (72.53%)	GSM3581020	Epicarp
EP_3_1	6.8	Paired	7.7G	44.18	92.3	51327504	37005506 (72.1%)	35645562 (69.45%)	GSM3581021	Epicarp
EP_3_2	7.6	Paired	8.04G	44.42	91.87	53567894	39313515 (73.39%)	37800834 (70.57%)	GSM3581022	Epicarp
EP_3_3	7.8	Paired	7.99G	44.53	91.75	53285840	39667085 (74.44%)	38260168 (71.8%)	GSM3581023	Epicarp
EP_4_1	8.4	Paired	8.03G	43.69	92.55	53543904	40962312 (76.5%)	39592087 (73.94%)	GSM3581024	Epicarp
EP_4_2	7.5	Paired	7.71G	43.76	91.3	51376358	38712583 (75.35%)	37385806 (72.77%)	GSM3581025	Epicarp
EP_4_3	7.3	Paired	7.7G	45.17	92.68	51309006	38519920 (75.07%)	37249669 (72.6%)	GSM3581026	Epicarp
EP_5_1	7.4	Paired	6.41G	44.33	89.11	42707518	30281426 (70.9%)	29303990 (68.62%)	GSM3581027	Epicarp
EP_5_2	6.4	Paired	6.73G	44.63	91.26	44854942	32849747 (73.24%)	31819548 (70.94%)	GSM3581028	Epicarp
EP_5_3	6.4	Paired	7.81G	44.22	91.51	52082040	38886011 (74.66%)	37677544 (72.34%)	GSM3581029	Epicarp
EP_6_1	7.8	Paired	8.01G	44.18	91.91	53430626	39349328 (73.65%)	38118394 (71.34%)	GSM3581030	Epicarp
EP_6_2	7.2	Paired	8G	44.35	91.41	53344338	38984479 (73.08%)	37740747 (70.75%)	GSM3581031	Epicarp
EP_6_3	6.7	Paired	7.9G	44.23	91.46	52655072	39069562 (74.2%)	37906707 (71.99%)	GSM3581032	Epicarp
AL_1_1	6.4	Paired	7.2G	44.57	92.31	48017132	36856574 (76.76%)	35728876 (74.41%)	GSM3581033	Albedo
AL_1_2	7.9	Paired	6.4G	44.6	92.08	42676132	32699791 (76.62%)	31582453 (74%)	GSM3581034	Albedo
AL_1_3	7.6	Paired	7.24G	44.76	92.34	48266214	36778487 (76.2%)	35600098 (73.76%)	GSM3581035	Albedo
AL_2_1	7.5	Paired	8.19G	45.05	91.67	54589638	40760109 (74.67%)	39655006 (72.64%)	GSM3581036	Albedo
AL_2_2	7	Paired	7.02G	45.4	92	46773600	35080122 (75%)	34093101 (72.89%)	GSM3581037	Albedo
AL_2_3	6.7	Paired	8.24G	45.3	91.96	54946558	41234109 (75.04%)	40110455 (73%)	GSM3581038	Albedo
AL_3_1	6.7	Paired	7.56G	45.57	91.37	50386566	36153339 (71.75%)	35179880 (69.82%)	GSM3581039	Albedo
AL_3_2	7.2	Paired	7.67G	43.08	91.73	51103134	36490988 (71.41%)	35504180 (69.48%)	GSM3581040	Albedo
AL_3_3	7.9	Paired	7.73G	45.2	91.8	51539758	37198121 (72.17%)	36202282 (70.24%)	GSM3581041	Albedo
AL_4_1	6.6	Paired	9.43G	44.87	92.61	62837140	43706630 (69.56%)	42563090 (67.74%)	GSM3581042	Albedo
AL_4_2	6.5	Paired	7.27G	45.36	91.63	48437484	36242613 (74.82%)	35321058 (72.92%)	GSM3581043	Albedo
AL_4_3	7	Paired	7.43G	45.11	92.4	49520012	37499914 (75.73%)	36501276 (73.71%)	GSM3581044	Albedo
AL_5_1	7.8	Paired	7.09G	44.5	91.56	47255256	35251434 (74.6%)	34420208 (72.84%)	GSM3581045	Albedo
AL_5_2	7.6	Paired	7.89G	44.44	91.15	52626680	38699976 (73.54%)	37758416 (71.75%)	GSM3581046	Albedo
AL_5_3	8.2	Paired	6.51G	44.62	91.95	43415178	32459250 (74.76%)	31671772 (72.95%)	GSM3581047	Albedo
AL_6_1	7.4	Paired	7.66G	44.61	92.94	51095852	38555401 (75.46%)	37620279 (73.63%)	GSM3581048	Albedo
AL_6_2	7.7	Paired	8.77G	44.33	93.32	58499262	44858162 (76.68%)	43757691 (74.8%)	GSM3581049	Albedo
AL_6_3	7.8	Paired	6.62G	44.25	93.47	44118938	33639225 (76.25%)	32843714 (74.44%)	GSM3581050	Albedo
SM_1_1	8.4	Paired	8.28G	44.98	91.7	55180648	41817148 (75.78%)	40693246 (73.75%)	GSM3581051	Segment membrane
SM_1_2	7.9	Paired	7.18G	44.79	90.88	47840002	36012887 (75.28%)	35025307 (73.21%)	GSM3581052	Segment membrane
SM_1_3	8.2	Paired	7.67G	44.72	91.43	51102360	38830750 (75.99%)	37791572 (73.95%)	GSM3581053	Segment membrane
SM_2_1	8	Paired	9.2G	45.06	92.2	61365126	45709235 (74.49%)	44408849 (72.37%)	GSM3581054	Segment membrane
SM_2_2	8.4	Paired	8.44G	45.91	91.99	56285078	42539322 (75.58%)	41354602 (73.47%)	GSM3581055	Segment membrane
SM_2_3	8.5	Paired	8.04G	45.93	92.2	53602616	40199469 (75%)	39123903 (72.99%)	GSM3581056	Segment membrane
SM_3_1	9.1	Paired	8.31G	45.61	91.75	55399450	40155146 (72.48%)	39062697 (70.51%)	GSM3581057	Segment membrane
SM_3_2	8.7	Paired	7.28G	44.47	92.1	48564052	35758991 (73.63%)	34826095 (71.71%)	GSM3581058	Segment membrane
SM_3_3	8.2	Paired	7.94G	44.67	92	52932872	38673778 (73.06%)	37659942 (71.15%)	GSM3581059	Segment membrane
SM_4_1	7.4	Paired	6.21G	46.22	90.98	41404494	29957420 (72.35%)	29223088 (70.58%)	GSM3581060	Segment membrane
SM_4_2	8.9	Paired	6.77G	44.25	91.46	45150108	33898882 (75.08%)	33091294 (73.29%)	GSM3581061	Segment membrane
SM_4_3	8.9	Paired	8G	45.17	91.35	53320578	39686991 (74.43%)	38710054 (72.6%)	GSM3581062	Segment membrane
SM_5_1	9.2	Paired	6.86G	46.11	91.26	45753488	33414737 (73.03%)	32616406 (71.29%)	GSM3581063	Segment membrane
SM_5_2	8.5	Paired	6.76G	45.58	91.63	45096224	33237302 (73.7%)	32447208 (71.95%)	GSM3581064	Segment membrane
SM_5_3	8.5	Paired	6.67G	45.82	91.42	44468660	32669828 (73.47%)	31872112 (71.67%)	GSM3581065	Segment membrane
SM_6_1	9	Paired	6.58G	45.75	95.5	43888890	33710438 (76.81%)	32930703 (75.03%)	GSM3581066	Segment membrane
SM_6_2	8.6	Paired	7.15G	46.64	95.94	47687968	36348235 (76.22%)	35442322 (74.32%)	GSM3581067	Segment membrane
SM_6_3	8.5	Paired	8.56G	46.18	95.96	57059680	43548585 (76.32%)	42523427 (74.52%)	GSM3581068	Segment membrane
JS_1_1	9.2	Paired	7.11G	44.96	91.38	47384024	35407896 (74.73%)	34434798 (72.67%)	GSM3581069	Juice sacs
JS_1_2	8.6	Paired	6.96G	44.7	91.63	46416938	34564741 (74.47%)	33613404 (72.42%)	GSM3581070	Juice sacs
JS_1_3	8.4	Paired	7.1G	45.43	91.61	47356512	35196276 (74.32%)	34238640 (72.3%)	GSM3581071	Juice sacs
JS_2_1	9.2	Paired	6.3G	45.76	92	42027790	31111449 (74.03%)	30269971 (72.02%)	GSM3581072	Juice sacs
JS_2_2	8.9	Paired	6.6G	45.63	92.05	43973404	32439775 (73.77%)	31552648 (71.75%)	GSM3581073	Juice sacs
JS_2_3	9.1	Paired	6.09G	45.73	91.75	40632284	29582071 (72.8%)	28823884 (70.94%)	GSM3581074	Juice sacs
JS_3_1	8.7	Paired	7.02G	45.46	92.34	46823064	34779287 (74.28%)	33844989 (72.28%)	GSM3581075	Juice sacs
JS_3_2	8.7	Paired	8.24G	45.49	92.5	54901224	40850785 (74.41%)	39789485 (72.47%)	GSM3581076	Juice sacs
JS_3_3	9.1	Paired	9.02G	45.91	92.35	60127274	43784660 (72.82%)	42661615 (70.95%)	GSM3581077	Juice sacs
JS_4_1	7.3	Paired	7.45G	45.07	93.68	49683580	38281123 (77.05%)	37405696 (75.29%)	GSM3581078	Juice sacs
JS_4_2	7.3	Paired	7.2G	44.88	93.51	48018170	37371494 (77.83%)	36513354 (76.04%)	GSM3581079	Juice sacs
JS_4_3	8.8	Paired	7.38G	44.86	93.47	49177396	38470541 (78.23%)	37601264 (76.46%)	GSM3581080	Juice sacs
JS_5_1	9.7	Paired	8.28G	46.1	92.05	55226718	41170157 (74.55%)	40225994 (72.84%)	GSM3581081	Juice sacs
JS_5_2	9.5	Paired	7.25G	45.93	91.49	48335866	35303777 (73.04%)	34491366 (71.36%)	GSM3581082	Juice sacs
JS_5_3	8.7	Paired	7.93G	45.56	91.69	52877896	38891288 (73.55%)	37971781 (71.81%)	GSM3581083	Juice sacs
JS_6_1	9.3	Paired	9.3G	44.67	93.21	61988428	48023590 (77.47%)	46884551 (75.63%)	GSM3581084	Juice sacs
JS_6_2	9.4	Paired	8.8G	45.63	93.19	58673662	45048041 (76.78%)	44015119 (75.02%)	GSM3581085	Juice sacs
JS_6_3	9.2	Paired	9.55G	44.8	92.91	63669642	49200769 (77.28%)	48083939 (75.52%)	GSM3581086	Juice sacs
